# Coexistence of Primary Sjögren’s Syndrome and Autoimmune Gastritis With Pernicious Anemia and Subacute Combined Degeneration of the Spinal Cord: Case Report and Literature Review

**DOI:** 10.3389/fimmu.2022.908528

**Published:** 2022-06-23

**Authors:** Hao-Su Zhan, Xin Yao, Hai-Yi Hu, Yan-Fei Han, Bing Yue, Li-Ying Sun, Yong-Jun Wang

**Affiliations:** ^1^ Department of Gastroenterology, Beijing Digestive Disease Center, Beijing Key Laboratory for Precancerous Lesion of Digestive Disease, National Clinical Research Center for Digestive Diseases, Beijing Friendship Hospital, Capital Medical University, Beijing, China; ^2^ Department of Critical Liver Diseases, Liver Research Center, Beijing Friendship Hospital, Capital Medical University, Beijing, China; ^3^ Liver Transplantation Center, National Clinical Research Center for Digestive Diseases, Clinical Diagnosis, Treatment and Research Center of Pediatric Liver Transplantation, Beijing Friendship Hospital, Capital Medical University, Beijing, China; ^4^ Clinical Center for Pediatric Liver Transplantation, Capital Medical University, Beijing, China; ^5^ Department of Neurology, Beijing Friendship Hospital, Capital Medical University, Beijing, China; ^6^ Department of Pathology, Beijing Friendship Hospital, Capital Medical University, Beijing, China

**Keywords:** primary Sjögren’s syndrome, autoimmune gastritis, pernicious anemia, subacute combined degeneration of the spinal cord, case report

## Abstract

**Background:**

Autoimmune gastritis (AIG) and Primary Sjögren’s syndrome (pSS) are both autoimmune diseases with low prevalence in China. Subacute combined degeneration (SCD) of the spinal cord is the most common neurological manifestation of vitamin B12 deficiency. Until now, a patient with pSS and complications of AIG including SCD has not been reported.

**Case Presentation:**

A 69-year-old woman presented with palpitations and symmetrical and progressive numbness in her hands and feet. The patient had a sense of stepping on cotton and could not write or walk without help. We reviewed the patient’s history and analyzed her blood tests, imaging, gastroscopic findings, and pathological results. The patient fulfilled the criteria of AIG, pSS, spinal cord SCD and early pernicious anemia (PA) simultaneously. Although pSS can lead to reduction of vitamin B12, this is the first overlapping case of pSS with spinal cord SCD. After symptomatic treatment, the patient returned to a normal life.

**Conclusions:**

This first report about the coexistence of pSS and complications of AIG including SCD and PA will promote a better understanding of the relationship between these diseases.

## Introduction

Autoimmune gastritis (AIG) is an increasingly prevalent, organ-specific, immune-mediated disorder characterized by destruction of gastric parietal cells, also called type A gastritis, resulting in the loss of intrinsic factor and reduced acid output. These alterations lead to malabsorption of iron, vitamin B12 and potentially other micronutrients (1). Subacute combined degeneration (SCD) of the spinal cord is the most common neurological manifestation of vitamin B12 deficiency and is usually secondary to AIG, but may also be seen in malnutrition syndromes such as chronic alcoholism, strict vegetarianism, gastrectomy, and also in nitrous oxide abuse. Primary Sjögren’s syndrome (pSS) is a systemic autoimmune disease characterized by lymphocytic infiltration of exocrine glands, mainly salivary and lacrimal glands (2). Chronic atrophic gastritis, including types A and B gastritis, is the most common presentation of gastrointestinal involvement in pSS (3), but cases of subacute combined degeneration in pSS have rarely been reported. As far as we know, there are no reports of patients with coexistence of AIG, spinal cord SCD and pSS. We report a case of AIG, associated with pSS, spinal cord SCD and PA, and discuss some aspects of this association.

## Case Presentation

A 69-year-old woman presented with palpitations and symmetrical and progressive numbness in her hands and feet; at the same time, the patient had a sense of stepping on cotton. When the patient visited the hospital, she could not write or walk without help.

**Figure 1:**
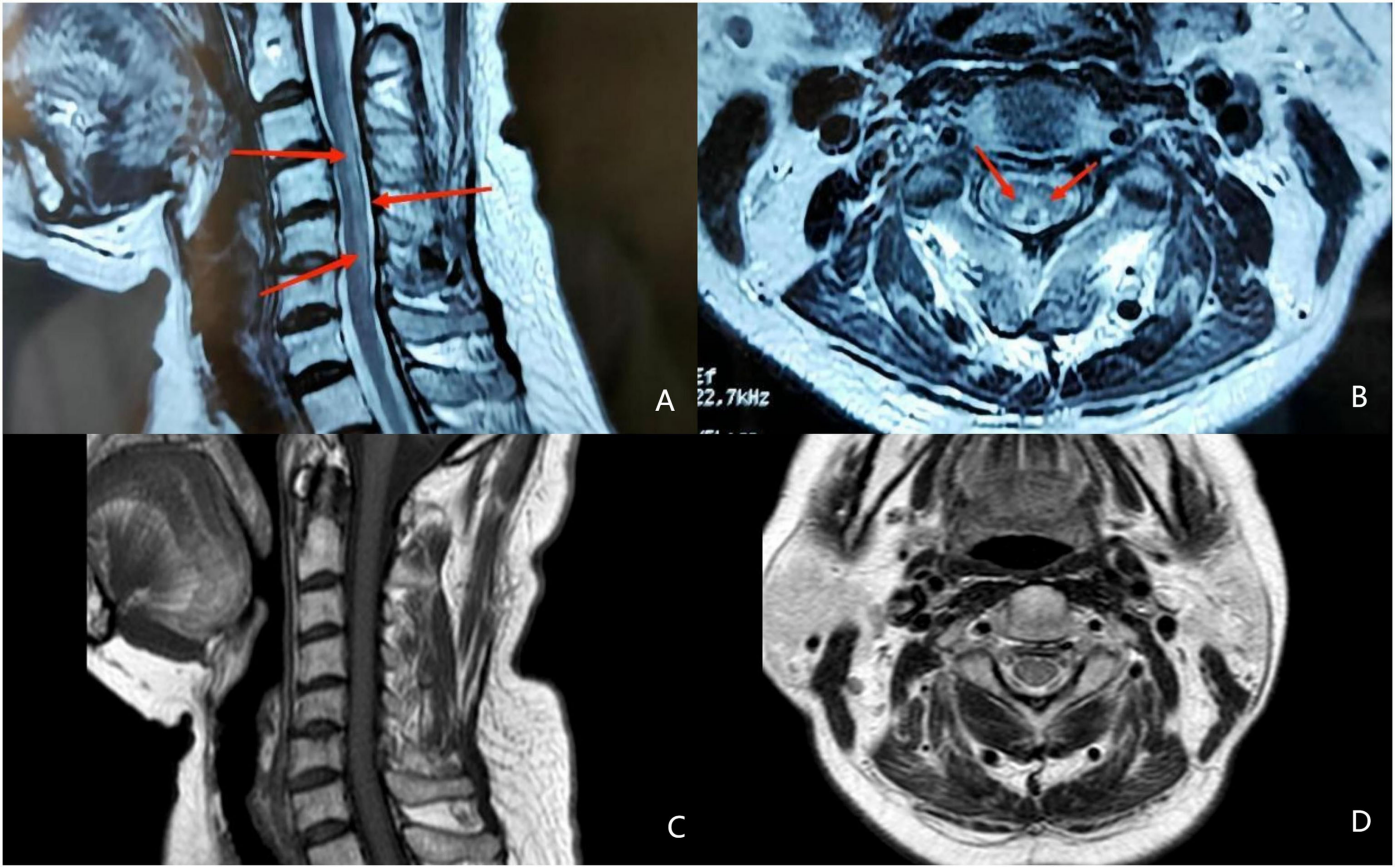
Magnetic resonance image (MRI) findings in the spinal cord. **(A)** abnormal longitudinally extensive T2 weighted hyperintensities involving the posterior columns (the red arrow) **(B)** inverted V or “rabbit ears” sign on cervical spinal (the red arrow). **(C)** abnormal hyperintensities involving the posterior columns disappeared in MRI after treament. **(D)** recovered MRI image on cervical spinal after treament.

The neurological examination showed a decrease of symmetrical short-glove, long-stocking-like acupuncture sensation. Her upper limb tendon reflex was reduced while her lower limb tendon reflex was active. Romberg’s test and bilateral Babinski tests were positive. The pathological reflex in cranial nerves was negative. Magnetic resonance imaging (MRI) of the brain revealed nonspecific cerebral white matter lesions. Cervical spinal cord MRI showed abnormal longitudinally extensive T2 weighted hyperintensities involving the posterior columns (**Figure 1A**), with inverted V or “rabbit ears” sign (**Figure 1B**). Electromyography (EMG) showed that sensory nerve conduction velocity of bilateral sural nerves was slowed down, suggesting peripheral neurogenic damage. Skin sympathetic response measurements showed a decrease in amplitude of the right median nerve.

Routine blood tests demonstrated macrocytic anemia: reduced red blood cell (RBC) count, increased mean corpuscular volume and mean corpuscular hemoglobin, normal mean corpuscular hemoglobin concentration, and elevated RBC volume distribution width, platelet and thrombocytocrit (**Table 1**). Other significant laboratory results revealed a reduced level of vitamin B12, elevated folic acid, normal ferritin, elevated erythrocyte sedimentation rate (ESR), elevated homocysteine (Hcy), elevated gamma-glutamyl transferase (GGT), decreased pepsinogen 1, nearly normal pepsinogen 2 and reduced pepsinogen 1/pepsinogen 2 ratio. Her gastrin level was elevated. Her parietal cell antibody, intrinsic factor antibody, antinuclear antibody, anti CENP B antibody and anti SSA60 were positive. The tumor marker CA72-4 was elevated. Her doctor administered symptomatic treatment and advised her to undergo gastroscopy and colonoscopy to rule out the possibility of digestive tract tumor, and the patient came to our hospital.

**Table 1:** Laboratory test results before and after treatment.

Items	Measured value		Reference range
	Before treatment	After treatment	
RBC (×1012/L)	3.33	4.07	3.5-5
Hb (g/L)	129	125	110-150
MCV (fL)	110.2	92.60	82-95
MCH (pg)	38.7	30.70	27-31
MCHC (g/L)	351	332	320-360
RBC volume distribution width (fl)	54.0	39.80	39.0-46.0
Platelet (×109/L)	388	231	100-350
Thrombocytocrit (%)	0.31	0.23	0.10-0.28
WBC (×109/L)	5.83	4.14	4-10
Vitamin B12 (pg/ml)	74.00	Medication	180-914
Folic acid (ng/ml)	>23.90	Medication	3.1-19.9
ESR (mm/h)	37	17	0-20
Homocysteine (μmol/L)	124.1	11.0	0-20
GGT (U/L)	55	24	7-45
A/G	1.49	1.72	1.5-2.5
Intrinsic factor antibody (Au/ml)	Positive (8.92)		Negative
Parietal cell antibody	+1:80	+1:80	Negative
ANA	+1:320 (centromere type)	+1:160 (centromere type)	Negative
Anti CENP B antibody	+++	++	Negative
Anti SSA60	+	Negative	Negative
CCP	Negative		Negative
RF	Negative	Negative	Negative
MTHFR	Mutant type (TT)		Negative
CYFRA21-1 (ng/ml)	3.47	3.84	0.1-3.3
CA72-4 (U/ml)	127.00	18.10	0-6.9
Alpha-fetoprotein (ng/ml)	13.70 (0.01-7)	10.08 (0.00-15)	In parentheses
C13 breath test	Negative	Negative	Negative
γ-globulin (%)	14.6	NA	9.1-24.0
β2-microglobulin (%)	6.3	NA	1.8-6.2
α1-globulin (%)	5.2	NA	2.2-4.8

RBC, red-cell count; Hb, hemoglobin; MCV, mean corpuscular volume; MCH, mean corpuscular hemoglobin; MCHC, mean corpusular hemoglobin concerntration; WBC, white blood cell; ESR, erythrocyte sedimentation rate; GGT, gamma-glutamyl transferase; ANA, anti-nuclear antibody; CCP, anti-cyclic citrullinated peptide antibody; RF, rheumatoid factors; MTHFR, Methylene tetrahydrofolate reductase; CA72-4, Cancer antigen CA72-4; NA, not available. + refers to a lighter degree, +++ refers to a heavier degree, and ++ refers to a degree between the two.

**Figure 2:**
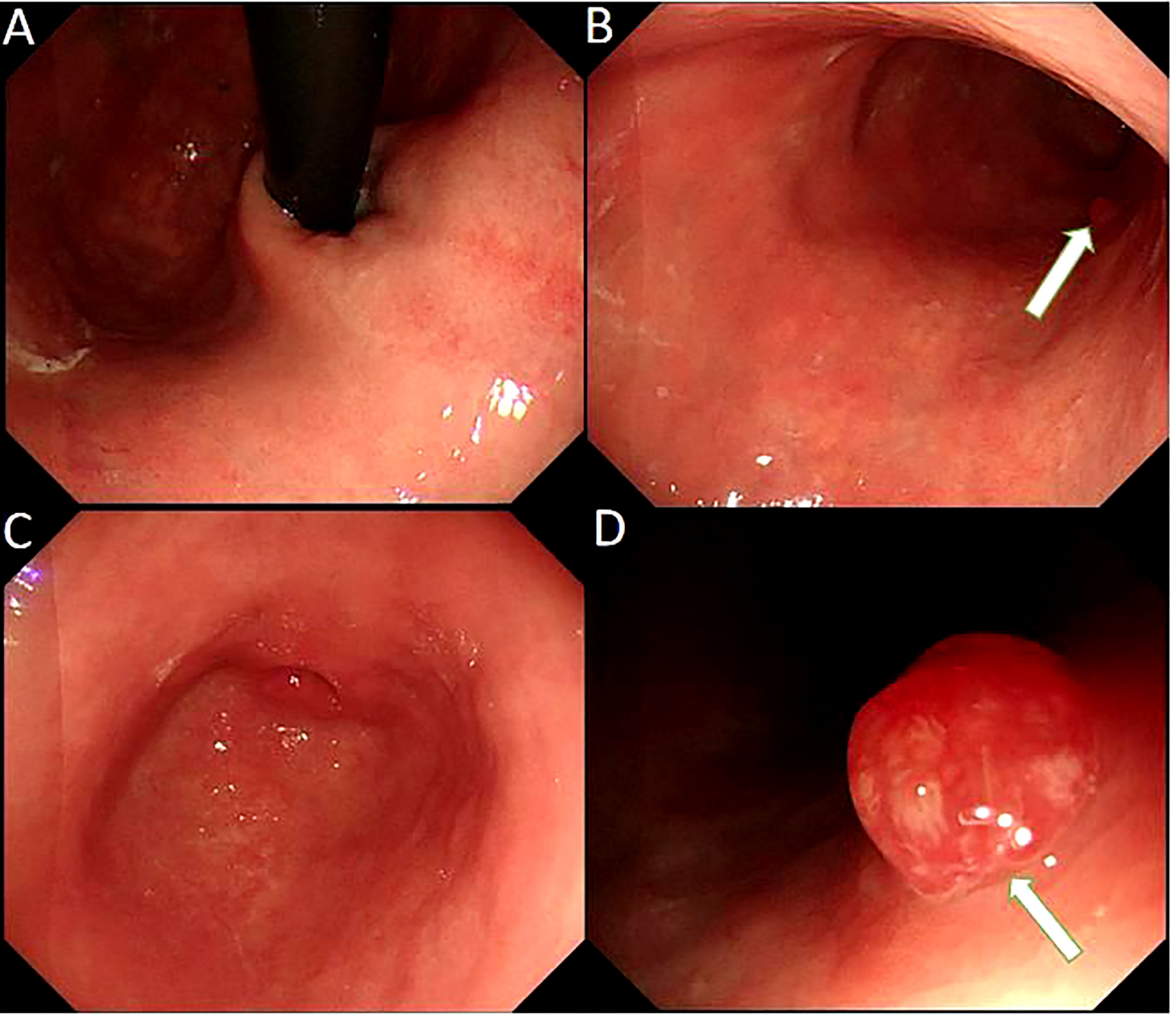
Gastroscope shows the atrophic gastritis in fundus **(A)** and corpus**(B)**, superficial gastritis in autrum **(C)** and a gastric polyp (arrow) in the corpus **(D)**.

**Figure 3:**
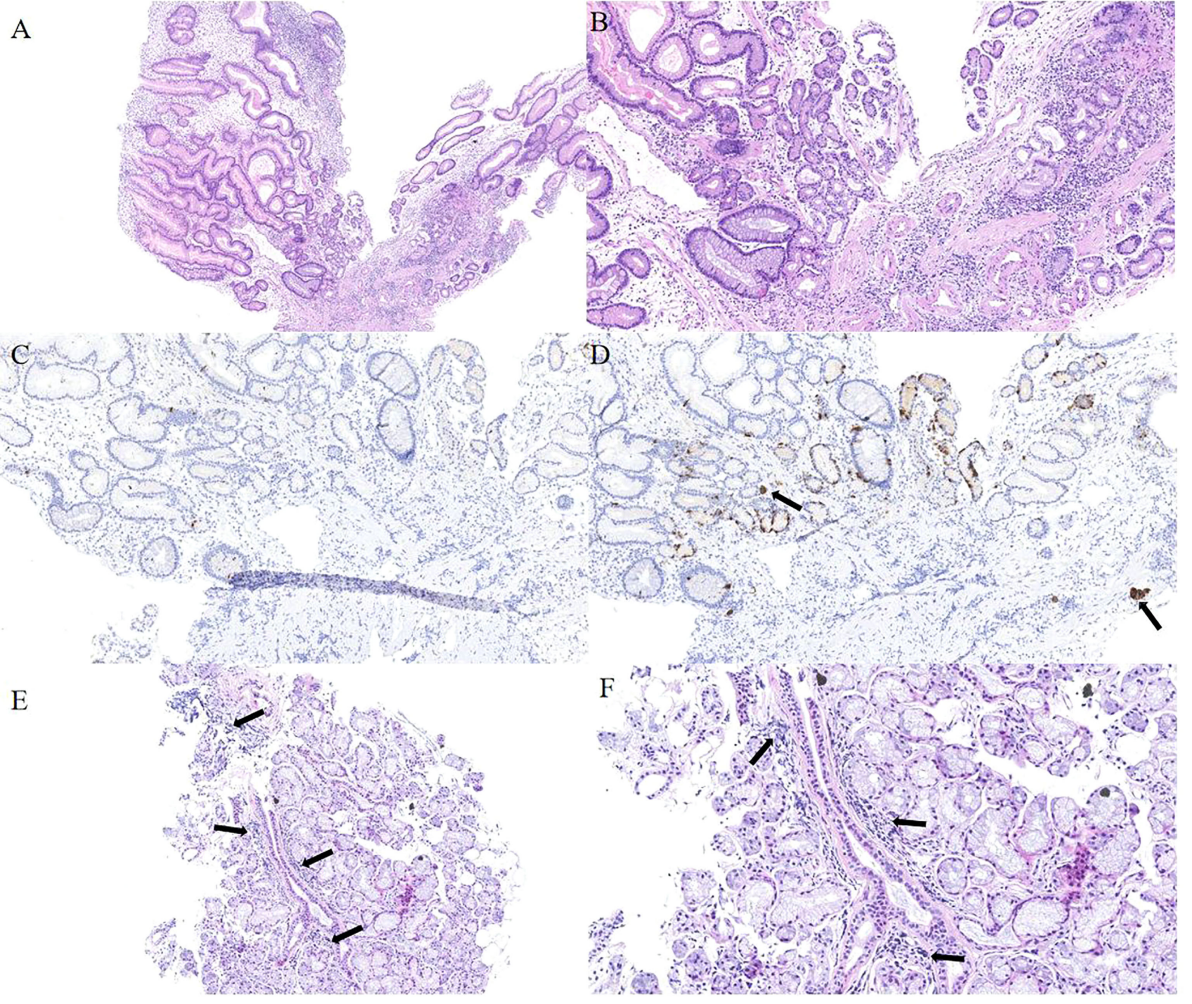
Histopathology of gastric mucosa and labial salivary gland. **(A)** Hematoxylin and eosin staining (40×) displays a hyperplastic polyp in the corpus. **(B)** Hematoxylin and eosin staining (100×) displays the background of the hyperplastic polyp: atrophy, intestinal metaplasia and pseudo-pyloric adenylation in the mucosa. **(C)**Immunohistochemical staining of gastrin is negative in pseudo-pyloric gland. **(D)** Staining with anti-chromogranin antibodies (CgA) (100×) depicts dark brown endocrine cells (arrow). **(E)** Hematoxylin and eosin staining (100×) displays no obvious atrophy in the acinus of the labial salivary gland, but small and numerous foci of lymphocytic aggregation was observed (arrow). **(F)** The arrow indicates the squeeze of the lymphocytes.

The patient had no digestive symptoms and has not taken any gastric medication (including proton pump inhibitors). Gastroscopy showed thin mucosa in the fundus and corpus, with a marked visible vascular pattern and fold atrophy. A red, 0.4×0.8 cm and Yamada type 2 polyp was found in the corpus (**Figure 2**). The polyp proved to be hyperplastic by histological examination. Biopsies of the fundus and corpus revealed atrophy, intestinal metaplasia and pseudopyloric adenylation, while pathological examination of the antrum indicated chronic inflammation. Immunostaining of gastric corpus with gastrin and anti-chromogranin antibodies revealed that pseudopyloric metaplasia had replaced oxyntic mucosa, and endocrine cells presented with a micronodular hyperplasia pattern (**Figure 3**). The characteristics under gastroscopy and pathological presentation were consistent with AIG. C 13 urea breath test for the diagnosis of *Helicobacter pylori* infection was negative. In view of the positive antinuclear antibody, we further reviewed her past medical history and found that she had dry eyes and dry mouth for more than 20 years. We performed ophthalmic examination and lip biopsy. Eye test showed ocular staining scores on two eyes were >5 Labial salivary gland histopathology indicated no obvious atrophy in the acinus, but small and numerous foci of lymphocytic aggregation were observed (50–80 lymphocytes/HPF; **Figure 3**), and focus score was 10.8. Based on her clinical manifestations of dry eyes and dry mouth, eyes test results, pathological results, and positive anti SSA60, the patient was finally diagnosed with pSS.

The patient was treated with 500 μg mecobalamin intramuscular injection three times a week, supplemented with vitamin B12, vitamin B2, folic acid and some traditional Chinese medicine as adjuvant therapy. After the diagnosis of pSS, the initial immunotherapy regimen with oral hydroxychloroquine and mycophenolate mofetil (MMF) was prescribed. However, the patient complained of sore eyes when taking hydroxychloroquine (0.2g bid). According to clinical experience and drug experiments (4–8), we finally adjusted the treatment regimen with total glucosides of paeony (0.6 g bid) and MMF (0.5g bid).

After 6 months of follow-up, her neurological symptoms were significantly relieved and the patient was able to return to a normal life. RBC count, vitamin B12 and Hcy were completely normal (**Table 1**). After 1 year of follow-up, her spinal cord imaging results were completely normal except for age-related degenerative changes (**Figures 1C, D**).

## Discussion and Conclusions

• Autoimmune gastritis is an organ-specific autoimmune disease characterized by an immune response that directly acts on parietal cells and intrinsic factor in the gastric body and fundus, resulting in gastric acid deficiency, hypergastrinemia, and insufficient production of intrinsic factor. The secretion of essential substances such as hydrochloric acid and intrinsic factor in the stomach is reduced in patients with AIG, leading to digestive dysfunction. From a clinical perspective, AIG can be reliably diagnosed by: (1) specific autoantibodies (parietal cell and intrinsic factor antibodies); (2) gastric mucosal function serology (declining pepsinogen 1, normal pepsinogen 2, reduced pepsinogen1/pepsinogen 2 ratio, and elevated gastrin-17); and (3) histology (using standard diagnostic biopsy sampling protocol) (9). AIG is typically restricted to the gastric corpus–fundus mucosa. Gastrin and pepsinogen levels can predict the level of atrophy. In advanced cases, the oxyntic epithelium is replaced by atrophic and metaplastic mucosa, creating the phenotypic background in which both gastric neuroendocrine tumors, hyperplastic polyps, and (intestinal-type) adenocarcinomas may develop (10). The most common clinical symptoms of AIG are megaloblastic anemia due to vitamin B12 deficiency and iron deficiency anemia due to iron malabsorption. In recent years, an increasing number of patients with AIG have had lack of vitamins and other micronutrients, such as vitamin C, vitamin D, folic acid and calcium. The occurrence of vitamin deficiency can lead to severe blood, nerve and bone diseases in AIG patients. Therefore, it also highlights the importance of the comprehensive evaluation of these patients. AIG is not uncommonly associated with other autoimmune conditions; some of which have gastrointestinal manifestations. AIG should be considered in patients with diagnosis of an autoimmune disease who present with megaloblastic anemia or iron deficiency anemia.

In addition, some studies have reported patients with PA may only present macrocytosis without anemia in the laboratory test for several months before the diagnosis of PA (11–13). According to the current diagnosis criteria, PA diagnosis may be delayed by 3 to 91 months in these patients. Macrocytosis might be a clue to the early diagnosis of PA. Therefore, although our patient presented with a macrocytosis and normal hemoglobin, an early PA could be diagnosed based on the macrocytosis, decreased vitamin B12, low RBC count and AIG.

There is limited guidance on the management of non-neoplastic complications of AIG/PA, including frequency of interval surveillance and monitoring for vitamin B12 deficiency and iron deficiency. Importantly, vitamin B12 deficiency can occur in the absence of anemia and if not addressed can lead to irreversible neurologic deficits. Parenteral rather than oral supplementation is preferred, especially if neurological symptoms are present. Appropriate supplementation of vitamin B12 and iron is important, along with establishing other etiologies for these not uncommon deficiencies based on the clinical scenario (1).

Although this patient had no digestive tract symptoms, such as abdominal distension and dyspepsia, the positive parietal cell and intrinsic factor antibodies, elevated gastrin, as well as pathological changes in the gastric mucosa indicated the presence of AIG, which may lead to impairment of vitamin B12 absorption, PA and other systemic lesions.

pSS is a chronic autoimmune disease of unknown etiology, mainly involving exocrine glands, especially salivary and lacrimal glands. The incidence is high in middle-aged and older women, and the male to female ratio is about 1:9. Although dryness (sicca) of the eyes and mouth are the classically described features, dryness of other mucosal surfaces and systemic manifestations, including fatigue and arthralgia, are common (14). Systemic features affect at least 70% and include inflammatory arthritis, skin involvement, hematological abnormalities, neuropathy, interstitial lung disease, and a 5–10% lifetime risk of B-cell lymphoma (15, 16). A variety of autoantibodies including SSA and SSB antibodies can be detected in the serum. The lymphocytic infiltration of the lacrimal and salivary glands, most commonly performed on labial salivary gland biopsies, is one of the hallmarks of the disease. The most characteristic feature of pSS on biopsy is focal lymphocytic sialadenitis, which is defined as the presence of dense aggregates (foci) of ≥50 mononuclear cells (mostly lymphocytes) (17). Eye tests including ocular staining scores(OSS) and Schirmer I test are widely used to diagnose pSS (18). SS itself is a rare disease that makes it difficult for doctors to identify the condition (19). According to the International Classification and Diagnostic Criteria for Sjögren syndrome 2017 (20), our patient scored 7 and could be diagnosed definitively for her positive anti-SSA60, OSS >5 of two eyes and salivary glands focus score>1. At the same time, the patient didn’t have any conditions listed as exclusion criteria. The patient didn’t have any other immunological characteristics of pSS and the European league against rheumatism Sjögren’s Syndrome Disease Activity Index (ESSDAI) of the patient was 0 (20).

Digestive involvement is frequent in pSS, including autoimmune disorders, such as chronic atrophic gastritis, esophageal motility dysfunction, lymphocytic colitis, primary biliary cholangitis and autoimmune hepatitis (2). Some pSS patients with chronic atrophic gastritis have parietal cell and/or intrinsic factor antibodies. The mechanism underlying the AIG in pSS may be reduced secretion of salivary epidermal growth factor (EGF). Deficiency of salivary gland-derived EGF in SS patients may cause impairment of gastric parietal cells, resulting in exposure of immunogenic cryptic antigens and loss of immunological self-tolerance (21). The condition of our patient helps us to understand the association of AIG and pSS. Additionally, according to previous studies, pSS and vitamin B12 deficiency have similar mechanisms in causing neuropathy, and some researchers have speculated that patients with pSS associated with vitamin B12 deficiency are more prone to neuropathy (22).

pSS-related atrophic gastritis and associated PA may require parenteral or sublingual vitamin B12 supplementation (2). Treatment of glandular lymphoproliferation and more severe extraglandular manifestations includes glucocorticoids, antimalarials (hydroxychloroquine), conventional nonbiological disease-modifying antirheumatic drugs such as methotrexate. pSS patients with sicca symptoms but without glandular enlargement or other organ involvement generally do not require systemic therapy other than secretagogues.

Spinal cord subacute combined degeneration is a rare neurodegenerative disease involving the posterior cord, lateral cord, and peripheral nerves of the spinal cord, characterized by demyelination of the lateral and dorsal columns of the spinal cord. The main symptoms are double lower limb or limb numbness, a feeling of stepping on cotton, walking instability, unable to stand smoothly after closing eyes, paralysis, limb tingling, which are often early manifestations of SCD. Cerebrospinal fluid examination is usually normal. MRI of the lower cervical and upper thoracic spinal cords often shows strip-shaped and spot-shaped lesions with low signal at T1 and high signal at T2. In EMG, both sensory and motor nerves are impaired; mainly with demyelinating lesions and some axonal injuries. Spinal cord SCD is caused by insufficient vitamin B12 due to its intake, absorption, combination, transport or metabolic disorders. AIG may lead to severe malabsorption of vitamin B12, and PA may occur in advanced stages. Other etiologies of vitamin B12 deficiency include insufficient intake due to long-term vegetarian diet, absorption disorders caused by gastrectomy, ileectomy, proton pump inhibitor (PPI) in long term treatment, alcoholism with atrophic gastritis, and snorting nitrous oxide. In patients with spinal cord SCD, further examination should be conducted to find out the potential cause and give effective treatment (23).

The patient had symptoms of progressive hand and foot numbness. The laboratory tests revealed decreased vitamin B12 and elevated Hcy. In addition, cervical spinal MRI showed inverted V or “rabbit ears” sign. These data were combined to confirm the diagnosis of spinal cord SCD. Symptomatic treatment should be carried out first, and the etiology should be identified simultaneously, followed by etiological treatment and auxiliary rehabilitation exercises.

The patient fulfilled the criteria of AIG, pSS, spinal cord SCD and PA. The presence of these diseases has been reported in the past, but to the best of our knowledge, this is the first overlapping case of pSS with spinal cord SCD. The etiology of spinal cord SCD is neurological lesions mainly caused by vitamin B12 deficiency. Some studies have confirmed that pSS can also lead to reduction of vitamin B12 (22, 24, 25), but the coexistence of the two diseases has not been reported until now. We supposed that spinal cord SCD in our patient was due to the reduction of vitamin B12 which may have been associated with pSS or AIG. Depending on the patient’s condition, vitamin B12 and folic acid supplementation should be used, as well as immunosuppressants for autoimmune diseases.

AIG is frequently found in association with thyroid disease, including Hashimoto’s thyroiditis, and type 1 diabetes mellitus. Other autoimmune conditions associated with AIG are Addison’s disease, chronic spontaneous urticaria, myasthenia gravis, vitiligo, and perioral cutaneous autoimmune conditions (26). Recently, Gonzalez et al. reported that the most common mucocutaneous immune-mediated disease in AIG was pSS in Chile (27). Because pSS has characteristics of both types of organ-specific and generalized autoimmune diseases, it occupies a central position among the other autoimmune diseases (28). Melchor et al. reported among 437 pSS patients in Spain that 4.8% presented with chronic atrophic gastritis (2). In Hungary, Pokorny et al. verified by histological examination that 31 of 44 pSS patients were diagnosed with chronic atrophic gastritis. Type A accounted for 29% of the chronic atrophic gastritis patients (29). In Italian pSS patients, parietal cell antibodies were positive in 10% of patients including 5% with atrophic gastritis (30). However, the association between AIG and pSS has not yet been reported in China. Furthermore, despite the high incidence of chronic atrophic gastritis in patients with pSS, PA is rarely reported in these patients in western countries (29, 31). Therefore, patients with pSS associated with PA and AIG are extremely rare in China.

AIG is silent and only becomes symptomatic 10–20 years later when the inflammatory gastric lesion progresses to chronic atrophic gastritis that manifests as either B12-deficient PA or iron deficiency anemia (32). Our patient had the symptoms of pSS for >20 years; therefore, it is difficult to identify which of AIG and pSS occurred earlier and was the initiating etiology of spinal cord SCD. The rare case presented the association of spinal cord SCD with AIG, PA and pSS. We need a more comprehensive understanding of these diseases and perform systematic examination in patients, and be vigilant during diagnosis and treatment to reduce missed diagnosis and give systemic treatment.

In patients whose first symptom is numbness in the limbs, we should be aware of the neurological symptoms of spinal cord SCD, and active etiological and symptomatic treatments should be initiated. Early, prompt and active treatment is critical to the prognosis of the disease, because the neurological changes are irreversible, and the examination to determine the specific etiology is equally important. None of SCD, AIG, PA and pSS is an isolated disease; therefore, we need to permanently establish the concept of integrated medical care and configure the relationship among different diseases. We suggest multidisciplinary cooperation to jointly evaluate the patient’s systemic condition and determine the specific etiology, screening for autoimmune diseases if necessary, so as to avoid missed diagnosis of hidden diseases that are not easy to discover. As the gastrointestinal tract is the frequently affected organ by many systematic diseases, doctors should be familiar with the gastrointestinal manifestations related to different diseases in their clinical practice and identify them as early as possible.

Although patients with pSS could develop AIG, as well as PA and SCD due to vitamin B12 deficiency, the coexistence of pSS and complications of AIG including macrocytosis (early PA) and SCD has not been reported in the literature. We report this case to promote a better understanding of the relationship among these diseases. We hope that the information about the current patient will be helpful for other researchers to realize the coexistence of pSS and multiple complications of AIG and elucidate the pathogenesis of the overlap.

## Data Availability Statement

The original contributions presented in the study are included in the article/supplementary material. Further inquiries can be directed to the corresponding author.

## Ethics Statement

The studies involving human participants were reviewed and approved by Medical Ethics Committee, Beijing Friendship Hospital, Capital Medical University. The patients/participants provided their written informed consent to participate in this study. Written informed consent was obtained from the individual(s) for the publication of any potentially identifiable images or data included in this article.

## Author Contributions

H-SZ and XY participated in manuscript writing, data collection and data analysis. H-YH, Y-FH, and BY participated in specialized guidance and data analysis. L-YS and Y-JW participated in clinical treatment and research revision. All authors contributed to the article and approved the submitted version.

## Funding

This work was supported by Beijing Science and Technology Planning Project (Z201100005620001).

## Conflict of Interest

The authors declare that the research was conducted in the absence of any commercial or financial relationships that could be construed as a potential conflict of interest.

## Publisher’s Note

All claims expressed in this article are solely those of the authors and do not necessarily represent those of their affiliated organizations, or those of the publisher, the editors and the reviewers. Any product that may be evaluated in this article, or claim that may be made by its manufacturer, is not guaranteed or endorsed by the publisher.
